# Vasculogenic mimicry score identifies the prognosis and immune landscape of lung adenocarcinoma

**DOI:** 10.3389/fgene.2023.1206141

**Published:** 2023-06-07

**Authors:** Weichang Yang, Zhouhua Li, Wenjun Wang, Juan Wu, Jinbo Li, Xiaotian Huang, Xinyi Zhang, Xiaoqun Ye

**Affiliations:** Department of Respiratory and Critical Care Medicine, The Second Affiliated Hospital of Nanchang University, Nanchang, Jiangxi, China

**Keywords:** vasculogenic mimicry, lung adenocarcinoma, prognosis, immune landscape, tumor microenvironment (TEM)

## Abstract

**Background:** Lung cancer has a high incidence and mortality rate worldwide. Vasculogenic mimicry (VM) is a specific modality of tumor angiogenesis that could potentially be a new target for tumor therapy. The purpose of this study was to explore the role of VM-related genes in assessing the prognosis and immune landscape of lung cancer.

**Methods:** VM-related genes were obtained from previous studies, and the expression data and clinical data of lung adenocarcinoma (LUAD) patients were obtained from the TCGA database and GEO database. We performed enrichment analysis of 24 VM-related genes and screened hub genes by constructing a protein–protein interaction network and using Cytoscape software. Subsequently, we developed the VM score based on univariate Cox regression analysis and Lasso analysis and validated the VM score on the GSE72094 dataset. In addition, we constructed a nomogram based on the VM score in the TCGA cohort. Finally, we explored the correlation between the VM score and the tumor microenvironment, immune cell infiltration, immune checkpoints, and drug sensitivity.

**Results:** Enrichment analysis revealed that VM-related genes were associated with the HIF signaling pathway and angiogenic pathway. We developed a VM score based on 3 genes (EPHA2, LAMC2 and LOXL2) in LUAD patients. Kaplan-Meier analysis showed that the VM score was associated with poor prognosis in LUAD patients. The receiver operating characteristic curve suggested that the VM score and nomogram are valid predictors for the overall survival of LUAD patients. The VM score was significantly correlated with immune cell infiltration, such as naïve B cells, neutrophils, and eosinophils, and there was a difference in the TME between the high VM score group and the low VM score group. LUAD patients in the high VM score group were more sensitive to antitumor drugs.

**Conclusion:** In summary, the VM score developed in this study is a valuable indicator for evaluating the prognosis and immune landscape of LUAD patients. VM may be a potential target for antitumor therapy in lung cancer.

## 1 Introduction

With the gradually increasing incidence of lung cancer, humans have had to endure great social and economic burdens resulting from lung cancer. According to a report from China, lung cancer has been the fastest growing malignancy in the last 30 years and is the leading cause of cancer death (Chen et al., 2022). Non-small cell lung cancer (NSCLC) is the main tissue type of lung cancer and includes lung adenocarcinoma (LUAD), lung squamous (LUSC) and large cell lung cancer (Thai et al., 2021). In recent years, great progress has been made in the treatment of lung cancer. The advent of immunotherapy and targeted therapy has greatly prolonged the survival of lung cancer patients, while the efficacy is limited due to drug resistance and recurrence (Hirsch et al., 2017). Therefore, it is necessary to explore potential therapeutic targets for lung cancer.

The mode of tumor blood vessel formation is complex; vasculogenesis and angiogenesis are the two main modes, while vasculogenic mimicry (VM) is a new mode of tumor circulation (Luo et al., 2020). VM is a novel angiogenic modality based on the melanoma model proposed by Maniotis et al. (1999) When the tumor volume is larger than 2 mm^3^, access to oxygen and nutrients by diffusion is not sufficient for the growth of tumor cells, and it is in this context that VM is formed (Treps et al., 2021). VM is considered a unique vascular model for tumors, unlike conventional angiogenesis, which is not dependent on endothelial cell production (Wechman et al., 2020). Studies have indicated that the presence of VM is characteristic of highly aggressive and metastatic tumor cells, which also suggests that VM is associated with poor prognosis in tumor patients (Cao et al., 2013; Zhang et al., 2019). In addition to melanoma, VM is also present in solid malignancies such as lung, breast and bladder cancers (Passalidou et al., 2002; Delgado-Bellido et al., 2017), which may explain the poor effectiveness of traditional antiangiogenic drugs. VM usually occurs in a hypoxic tumor microenvironment, and the main signaling pathways associated with VM are the VE-cadherin (VE-Cad), Notch and HIF pathways (Delgado-Bellido et al., 2017; Valdivia et al., 2019). These signaling pathways are all interconnected, with VE-Cad being the factor that intersects most with other signaling pathways (Paulis et al., 2010). Some progress has been made in recent years on the mechanisms of VM formation in lung cancer. VE-cadherin (VE-Cad) has been reported to promote VM formation in lung cancer and to provide the blood supply required for lung cancer cell proliferation and invasion (Ding et al., 2018). In addition, previous studies have confirmed the role of DKK1, MMP2, MMP13 and ERβ in promoting VM formation in NSCLC (Yao et al., 2016; Li et al., 2017; Yu et al., 2019). Encouragingly, a growing number of studies have identified relevant genes and pathways involved in VM in lung cancer (Xia et al., 2019; Li et al., 2021; Shi et al., 2022; Zhang et al., 2022), which provides more evidence to clarify the mechanism of VM. However, no studies have reported the interaction of VM-related genes in lung cancer.

In this study, we obtained VM-related genes by a literature review and explored the relevant functions and expression of VM-related genes in LUAD samples based on The Cancer Genome Atlas (TCGA) database. Furthermore, we established VM scores based on univariate Cox regression analysis and Lasso regression analysis and evaluated the correlation of VM scores with prognosis and immune cell infiltration. Finally, we compared the correlation of the VM score with the sensitivity of some common antitumor drugs to provide new ideas for the clinical treatment of lung cancer.

## 2 Materials and methods

### 2.1 Data acquisition

**FIGURE 1:**
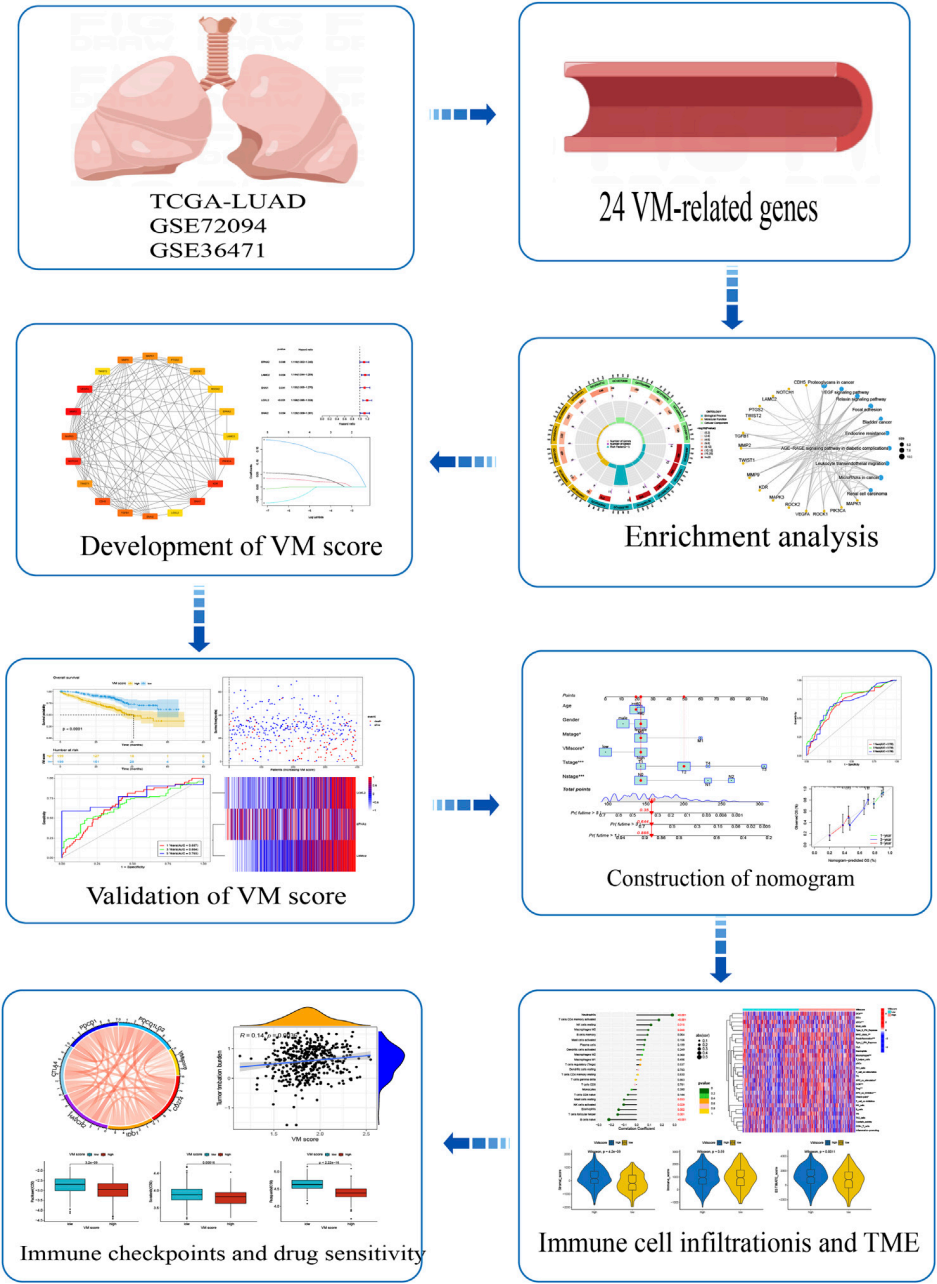
Flow chart of this study.

The expression data and clinical data of LUAD patients were downloaded from the TCGA website (https://portal.gdc.cancer.gov/), and incomplete data were excluded; consequently, 460 patients with LUAD were included in this study. The GSE72094 dataset was downloaded from the Gene Expression Omnibus (GEO) website (https://www.ncbi.nlm.nih.gov/geo/) and ultimately included 390 LUAD patients in GSE72094 and 115 LUAD patients in GSE36471. We obtained 24 VM-related genes from the earlier literature in PubMed and mainly referenced the VM-related genes obtained by Wang et al. (Ruf et al., 2003; Yu et al., 2008; Liu et al., 2011; Lu et al., 2013; Sun et al., 2013; Kim et al., 2019; Wechman et al., 2020; Zhang et al., 2020; Zhu et al., 2020; Herrera-Vargas et al., 2021; Mo et al., 2021; Morales-Guadarrama et al., 2021; Lee et al., 2022; Wang et al., 2022) The literature sources for all VM-related genes are listed in Supplementary Table S1. The flow chart of this study is shown in Figure 1.

### 2.2 Functional analysis of VM-related genes

To explore the functions and pathways of 24 VM-related genes, the “clusterProfiler” package was used for Gene Ontology (GO) enrichment analysis and Kyoto Encyclopedia of Genes and Genomes (KEGG) enrichment analysis. The GO enrichment included three categories: biological process (BP), molecular function (MF), and cellular component (CC). The definitions of BP, MF and CC are as follows. BP: the pathways and larger processes to which gene product activity contributes; MF: the molecular activities of individual gene products; and CC: where the gene products are active (http://geneontology.org/docs/go-annotations/). The threshold values of the *p*-value and the q-value were set to 0.05. Additionally, gene set enrichment analysis (GSEA) was used to explore the pathway differences between the high- and low- VM score groups.

### 2.3 Screening of hub genes

Twenty-four VM-related genes were uploaded to the STRING website (https://string-db.org/) to construct the protein–protein interaction (PPI) network. The interaction score was 0.400, and disconnected nodes were removed from the network. The cytoHubba plugin of Cytoscape software (version 3.9.1) was utilized to calculate the betweenness (BC) values of VM-related genes. Hub genes were selected according to the BC values.

### 2.4 Construction and validation of the VM score

To screen for prognostic genes, the “survival” package was used to perform univariate Cox regression analysis on VM-hub genes based on the TCGA cohort. In addition, Lasso regression was utilized to further screen genes to calculate the VM score. LUAD patients were classified into high- and low-risk groups according to the median VM score. The VM score was validated in the GSE72094 and GSE36471 datasets.

### 2.5 Development of nomogram based on VM score

We analyzed the correlation between the VM score and clinical characteristics; subsequently, the VM score and clinical characteristics (age, sex, T stage, N stage, and M stage) were included in multiple linear regression analysis, and the “rms” package was used to construct a nomogram. In addition, the “timeROC” package was used to predict the overall survival (OS) of LUAD patients at 1, 3 and 5 years.

### 2.6 Immune related profile analysis based on the VM score

The “CIBERSORT” package and “GSVA” package were used to analyze the correlation between the VM score and immune cell infiltration. We then compared the difference in the tumor microenvironment (TME) and VM score; the TME scores were downloaded from the TIMER website (http://timer.comp-genomics.org/). In addition, we explored the correlation between VM scores and immune checkpoints and tumor mutation burden (TMB). The “pRRophetic” package was utilized to explore the differences in drug sensitivity between the groups with high and low VM scores.

### 2.7 Statistical analysis

R (version 4.2.2) was used to perform all statistical analyses. The Wilcoxon test, Spearman correlation analysis and survival analysis were used in this study. *p* < 0.05 was considered statistically significant.

## 3 Results

### 3.1 VM-related gene expression and enrichment analysis

**FIGURE 2:**
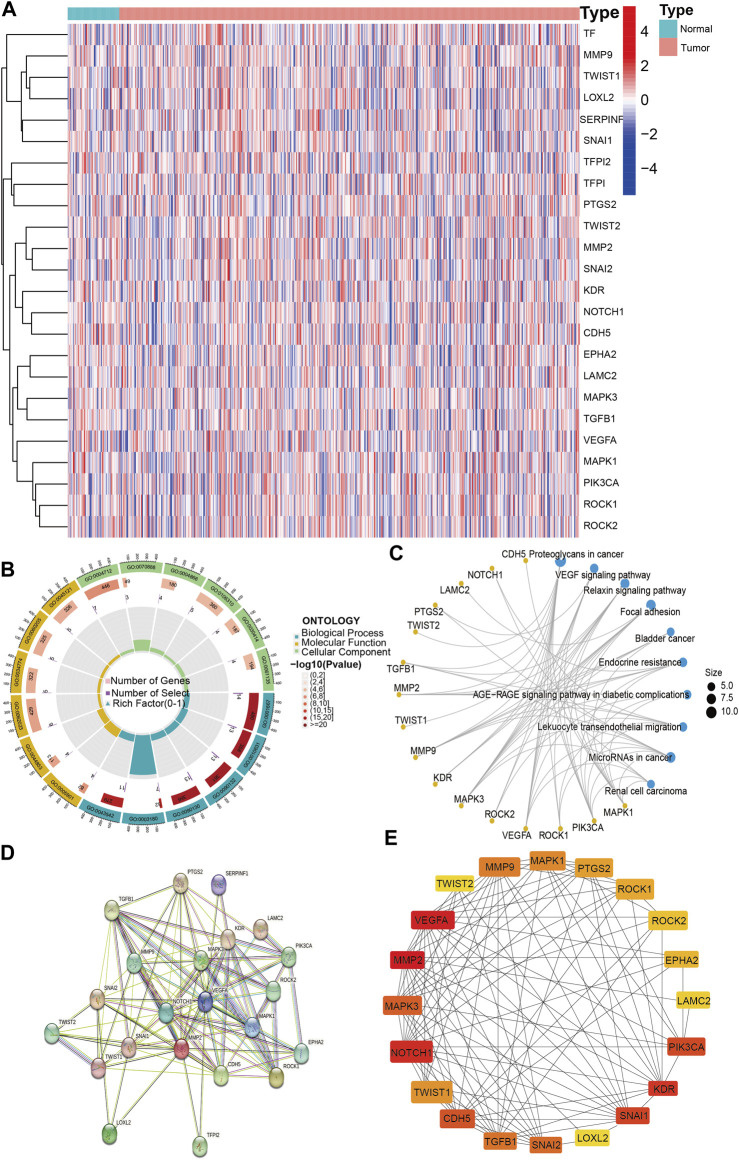
VM-related genes expression and Hub genes screening. **(A)**. The expression of VM-related genes between tumor and normal. **(B)**. GO enrichment analysis of VM-related genes. **(C)** KEGG enrichment analysis of VM-related genes. **(D)** PPI network of VM-related genes. **(E)** Top 20 hub genes of VM-related genes. VM: vasculogenic mimicry. GO: Gene Ontology. KEGG: Kyoto Encyclopedia of Genes and Genomes. PPI: protein–protein interaction. Molecular Function: the molecular activities of individual gene products. Cellular Component: where the gene products are active. Biological Process: the pathways and larger processes to which that gene product’s activity contributes.

To explore the expression and function of VM-associated genes, we analyzed the expression of VM-associated genes in lung adenocarcinoma and normal tissues and performed enrichment analysis. The results showed that the differentially expressed genes (*p* < 0.05) were TFPI, SERPINF1, TF, VEGFA, NOTCH1, CDH5, KDR, PTGS2, MMP9, TWIST1, MMP2, LOXL2, TFPI2 and TWIST2 (Figure 2A). In addition, we performed GO enrichment analysis for 24 VM-associated genes, and the results were as follows (Figure 2B). BP: amoeboid-type cell migration, epithelial cell migration, and epithelium migration; CC: caveola, plasma membrane raft and collagen-containing extracellular matrix; and MF: protein serine/threonine/tyrosine kinase activity, E-box binding, and endopeptidase inhibitor activity binding. The KEGG enrichment analysis showed that the key pathways were proteoglycans (PGs) in cancer, the vascular endothelial growth factor (VEGF) signaling pathway and the relaxin signaling pathway (Figure 2C). The results of VM-related gene enrichment analysis are shown in Supplementary Table S2 and Supplementary Table S3. The above results confirmed that VM-related genes were differentially expressed in lung adenocarcinoma and that VM-related genes were mainly involved in angiogenesis-related pathways.

### 3.2 PPI network and hub genes

To screen the hub genes, we imported 24 VM-related genes into the STRING website, containing 105 edges and 24 nodes; disconnected nodes were removed, and 22 VM genes were screened (Figure 2D). We further filtered the hub genes based on cytoHubba in Cytoscape software. Ultimately, 20 VM-related hub genes were screened based on BC values (Figure 2E).

### 3.3 Development and validation of the VM score

**FIGURE 3:**
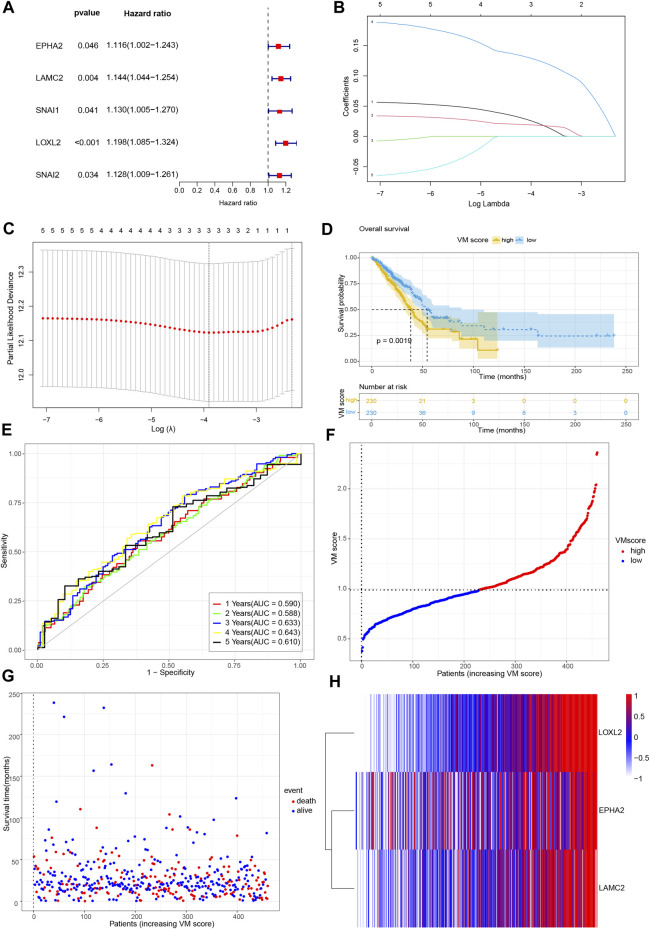
Development of VM score. **(A)** Forest plot of univariate Cox regression analysis of VM-related genes. **(B)** LASSO coefficient profiles of 5 genes. **(C)** Cross-validation of tuning parameter selection in the LASSO Cox regression. **(D)** KM analysis of overall survival in high VM score group and low VM score group based on TCGA database. **(E)** Time-dependent ROC curve of VM score. **(F)** VM score distribution, **(G)** survival status of patients, and **(H)** heatmap of VM-related genes distribution in TCGA cohort. VM: vasculogenic mimicry. TCGA: the Cancer Genome Atlas. ROC: Receiver operating characteristic.

Previous results screened the 20 hub genes. To identify the prognostic value of VM-related genes in patients with lung adenocarcinoma, we performed univariate Cox regression analysis on 20 VM-hub genes (Table 1) and screened for 5 prognostic genes (Figure 3A). Subsequently, LASSO Cox regression analysis was performed on 5 prognostic genes, and 3 genes were finally screened: EPHA2, LAMC2 and LOXL2 (Figures 3B,C). The VM scores were calculated for each sample based on Lasso regression coefficients: VM score = 0.028*EPHA2+ 0.019* LAMC2+ 0.131* LOXL2. A total of 460 samples were divided into two groups (high VM score and low VM score) according to the median VM score. Kaplan‒Meier (KM) analysis showed that the low VM score group had better OS than that of the high VM score group (*p* < 0.05) (Figure 3D). A receiver operating characteristic (ROC) curve was used to evaluate the predictive power of the VM score based on the TCGA database, and the areas under the curve (AUCs) at 1 year, 2 years, 3 years, 4 years and 5 years were 0.590, 0.588, 0.633, 0.643, and 0.610, respectively (Figure 3E). Internal validation was performed in the TCGA cohort (Figures 3F–H).

**TABLE 1:** The univariate Cox regression analysis of VM-related genes.

Gene	HR	95% CI	*p*-value
MAPK1	0.97	(0.80–1.18)	0.76
PIK3CA	1.01	(0.83–1.22)	0.94
ROCK1	0.96	(0.81–1.15)	0.66
VEGFA	1.03	(0.92–1.16)	0.59
NOTCH1	0.93	(0.81–1.07)	0.29
ROCK2	0.98	(0.82–1.17)	0.82
MAPK3	0.95	(0.79–1.14)	0.59
EPHA2	1.12	(1.00–1.24)	0.046
LAMC2	1.14	(1.04–1.25)	0.004
CDH5	0.93	(0.81–1.06)	0.27
KDR	0.92	(0.83–1.02)	0.10
PTGS2	1.05	(0.98–1.12)	0.14
MMP9	1.02	(0.94–1.11)	0.64
SNAI1	1.13	(1.00–1.27)	0.041
TWIST1	1.05	(0.98–1.13)	0.20
MMP2	0.96	(0.85–1.07)	0.42
LOXL2	1.20	(1.08–1.32)	<0.001
SNAI2	1.13	(1.01–1.26)	0.034
TGFB1	1.00	(0.87–1.16)	0.97
TWIST2	1.11	(0.99–1.24)	0.085

**FIGURE 4:**
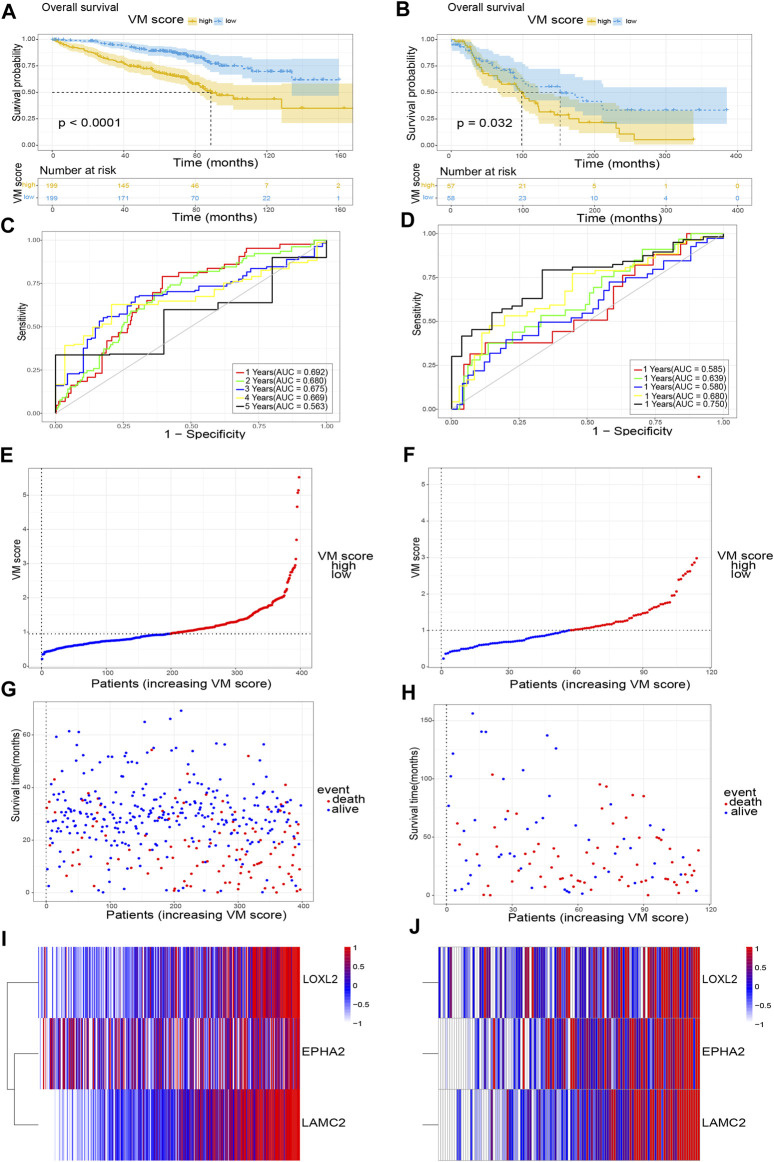
Validation of VM score. **(A)**, **(C)**, **(E)**, **(G)** and **(I)** The validation of VM score in GSE72094; **(B)**, **(D)**, **(F)**, **(H)** and **(J)** The validation of VM score in GSE36471. **(A)**, **(B)** KM analysis of OS. **(C)**, **(D)** Time-dependent ROC curve of VM score. **(E)**, **(F)** VM score distribution, **(G)**, **(H)** survival status of patients, and **(I)**, **(J)** heatmap of VM-related genes distribution. VM: vasculogenic mimicry. KM: Kaplan-Meier. ROC: Receiver operating characteristic.

**FIGURE 5:**
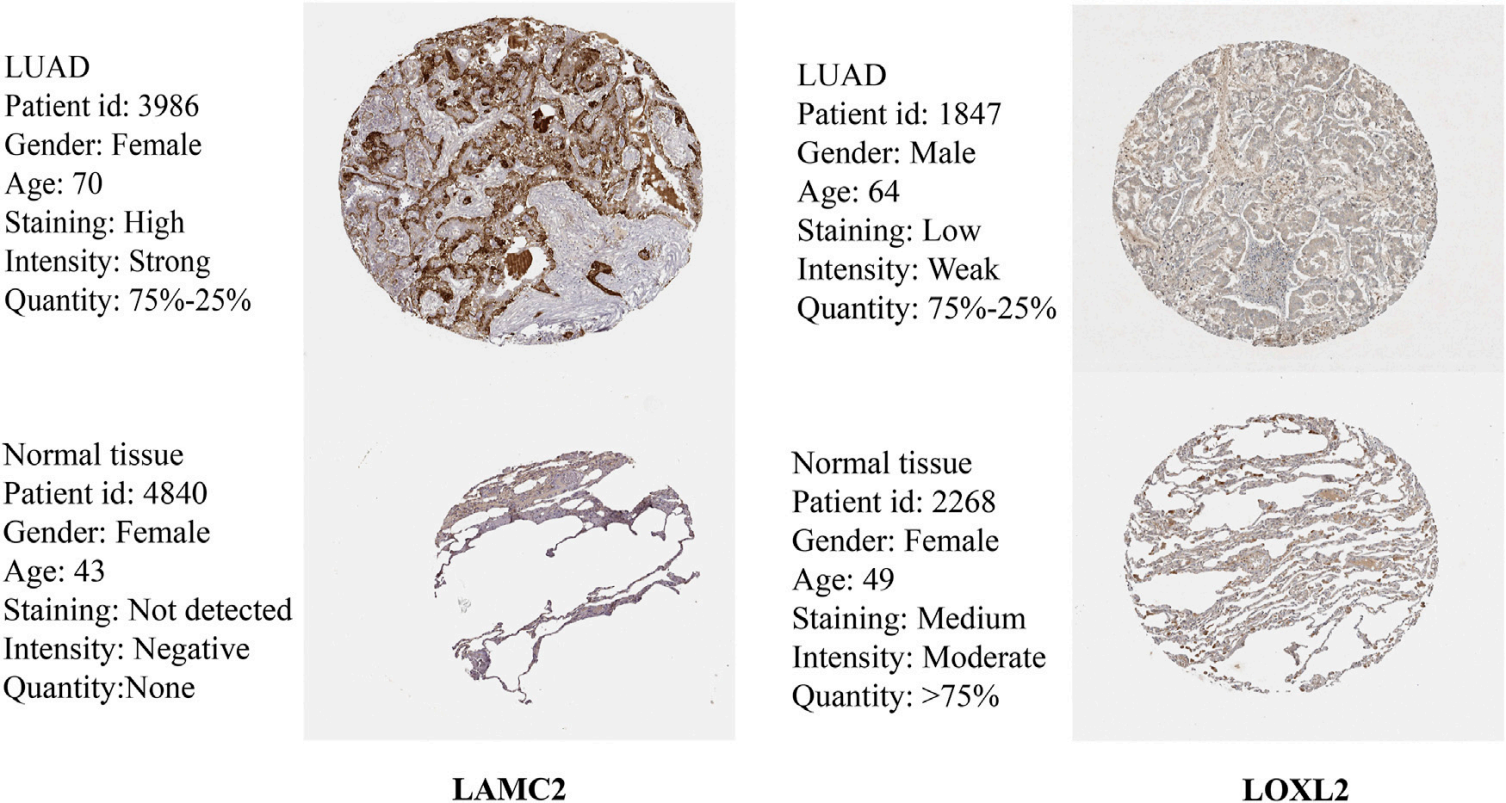
Expression of LAMC2 and LOXL2 in LUAD and normal tissues.

In addition, we further validated the stability of the VM score in the GSE72094 and GSE36471 datasets. Similarly, all samples were divided into two groups (high VM score group and low VM score group). KM analysis showed that the OS of the low VM score group was higher than that of the high VM score group (*p* < 0.05) (Figures 4A,B). The AUCs at 1 year, 2 years, 3 years, 4 years and 5 years were 0.692, 0.680, 0.675, 0.669 and 0.563, respectively, in the GSE72094 dataset (Figure 4C) and 0.585, 0.639, 0.580, 0.680, and 0.750, respectively, in the GSE36471 dataset (Figure 4D). External validation was performed in the GSE72094 dataset (Figures 4E,G,I) and the GSE36471 dataset (Figures 4F,H,J). The immunohistochemistry results of LAMC2 and LOXL2 based on The Human Protein Atlas are shown in Figure 5. These results confirmed that the VM score was a valuable indicator to identify the prognosis of patients with lung adenocarcinoma.

### 3.4 Development of a nomogram based on VM score

**FIGURE 6:**
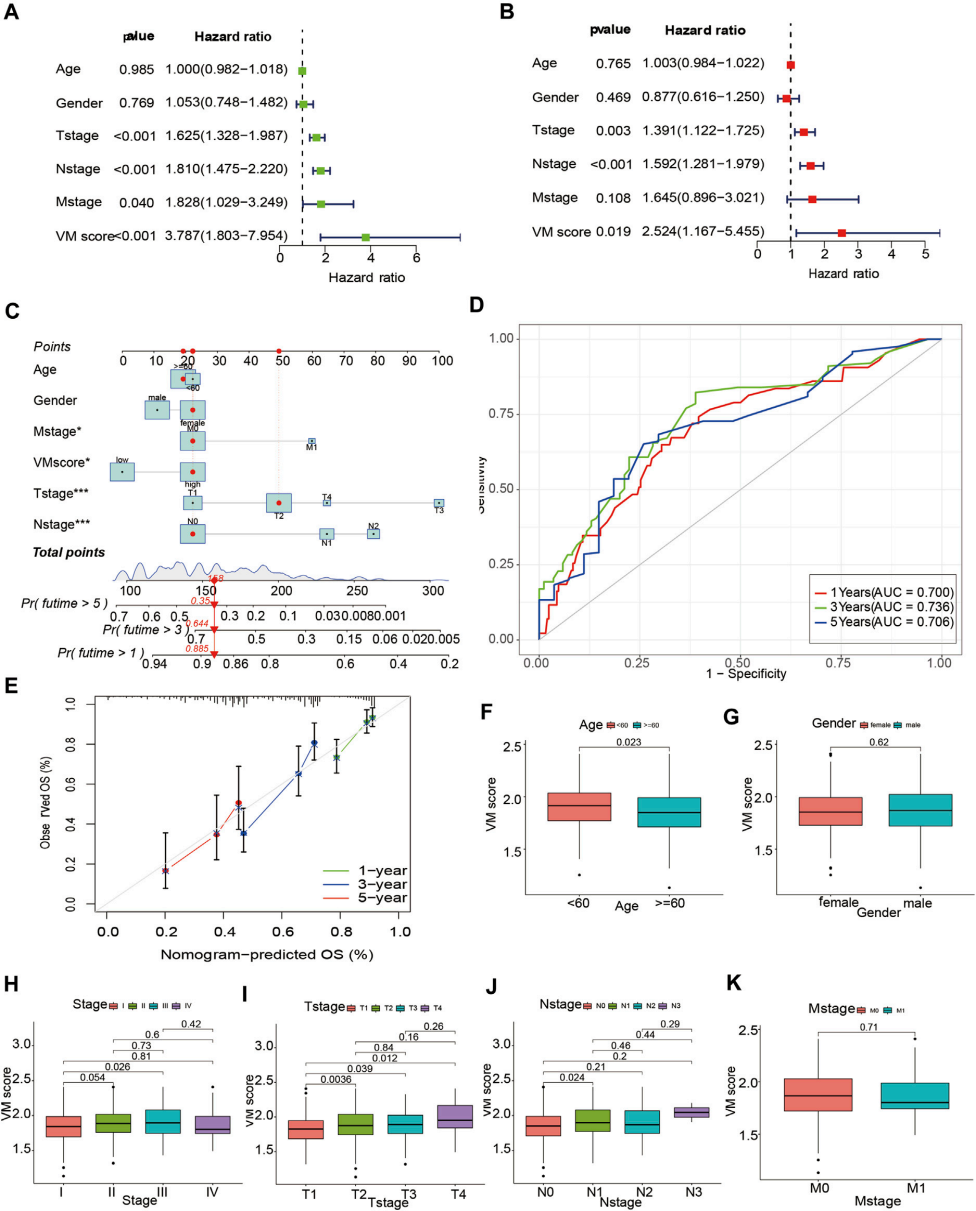
Development of a nomogram based on VM score. **(A)** Forest plot of univariate Cox regression analysis. **(B)** Forest plot of multivariate Cox regression analysis. **(C)** Nomogram for LUAD patients based VM score. **(D)** Time-dependent ROC curve of nomogram. **(E)** Calibration curves of nomogram model. **(F–K)**: Box plots of VM score with clinical factors. **(F)** Age, **(G)** Gender, **(H)** Stage, **(I)** T-stage, **(J)** N-stage, **(K)** M-stage. VM: vasculogenic mimicry. LUAD: lung adenocarcinoma.

To apply the VM score to clinical work with lung adenocarcinoma patients, we combined the VM score with clinical information to develop a nomogram. We explored the associations between the VM score and clinical information. The results showed that a high VM score was associated with worsening age (Figure 6F), T stage (Figure 6I) and stage (Figure 6H), suggesting that a high VM score indicated a worse prognosis in LUAD patients. However, no significant differences were observed in sex (Figure 6G), N stage (Figure 6J), M stage (Figure 6K) and the VM score. To further explore the predictive ability of the VM score on OS in LUAD patients, we performed univariate and multivariate Cox analyses for the VM score and clinical information (T stage, N stage, M stage, age and sex). The univariate Cox analysis indicated that the VM score was associated with prognosis in LUAD patients (HR = 3.787, 95% CI = 1.803–7.954, *p* < 0.001) (Figure 6A); the multivariate Cox analysis indicated that the VM score was an independent predictor for LUAD patients (HR = 2.524, 95% CI = 1.167–5.455, *p* < 0.05) (Figure 6B). Furthermore, we developed a nomogram to predict the survival probability of LUAD patients (Figure 6C). The 1-year, 3-year and 5-year AUCs of the nomogram were 0.700, 0.736, and 0.706, respectively (Figure 6D). The calibration curve indicated that the actual probabilities were in accordance with the predicted probabilities (Figure 6E). These results suggested that the nomogram based on the VM score could well predict the survival status of lung adenocarcinoma patients.

### 3.5 GSEA based on VM score

**FIGURE 7:**
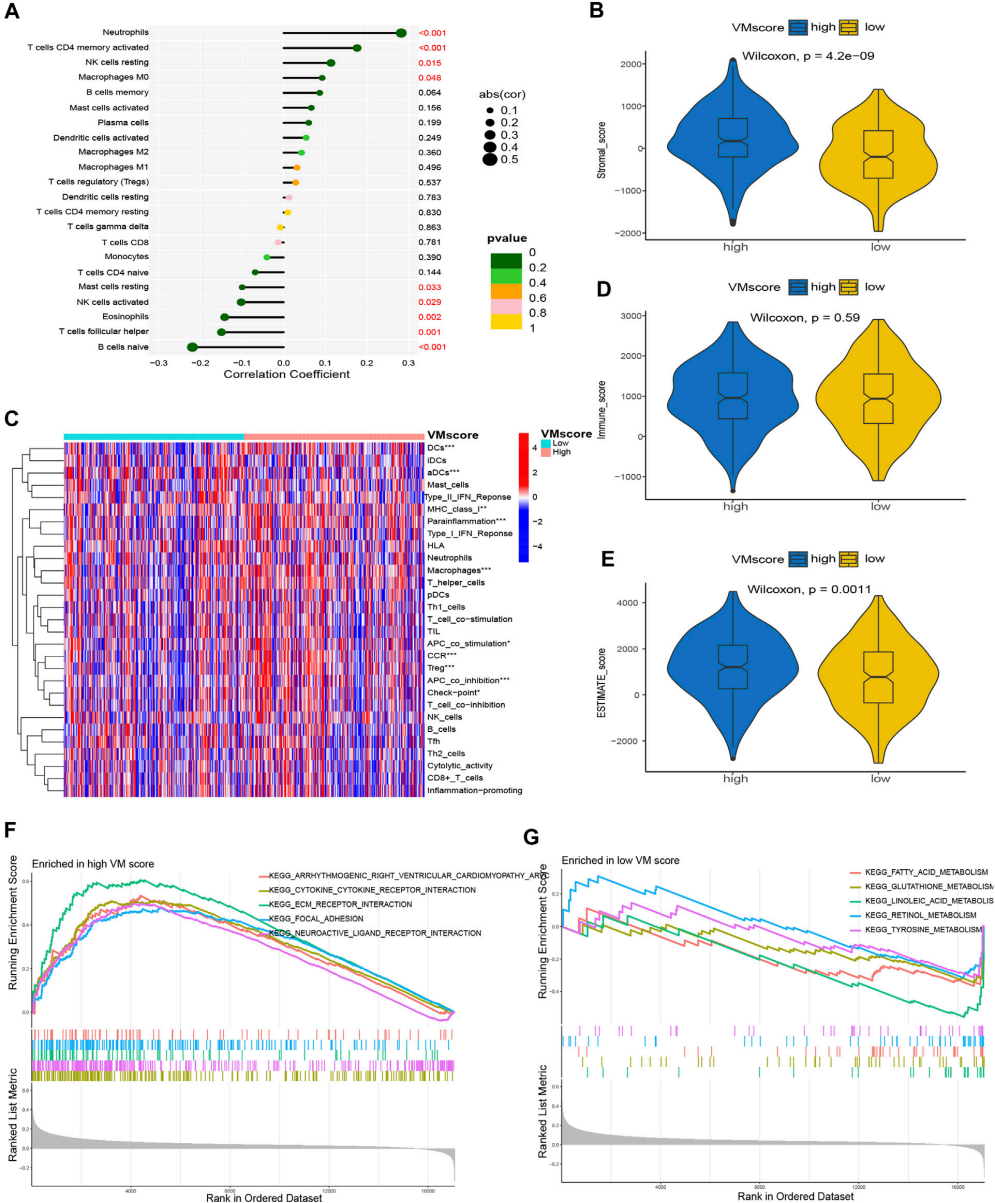
Correlation between VM score and immune landscape. **(A)** Correlation of VM score with immune cell infiltration based on CIBERSORT. **(C)** Differences in immune cell infiltration in high VM score and low VM score based on ssGSEA. **(B)**, Dand **(E)** The difference in TME between the high VM score and low VM score groups. **(B)** Stromal score, **(D)** Immune score, **(E)** ESTIMATE score. **(F)** GSEA in high VM score group. **(G)** GSEA in low VM score group. VM: vasculogenic mimicry. GSEA: gene set enrichment analysis.

Previous studies confirmed the prognostic status of the high and low VM score groups. To further explore the differences in enrichment pathways between high and low VM scores, GSEA was performed between the high and low VM score groups. The results showed that the pathways associated with the high VM score group were arrhythmogenic right ventricular cardiomyopathy, cytokine‒cytokine receptor interaction and ECM–receptor interaction (Figure 7F); the pathways associated with the low VM score group were fatty acid metabolism, glutathione metabolism and linoleic acid metabolism (Figure 7G).

### 3.6 VM score and immune landscape

To further assess the correlation between the VM score and immune cell infiltration, we used the CIBERSORT and ssGSEA algorithms for analysis. The CIBERSORT results showed that neutrophils, activated memory CD4 T cells, resting NK cells and M0 macrophages were positively correlated with the VM score; resting mast cells, activated NK cells, eosinophils, follicular helper T cells and naïve B cells were negatively correlated with the VM score (Figure 7A). The results of ssGSEA showed that DCs, aDCs, MHC class I, parainflammation, macrophages, APC costimulation, CCR, Treg, APC coincubation and checkpoint were significantly different between the two groups (Figure 7B). In addition, we assessed the difference in the TME between the two groups. The results showed that the stromal score (Figure 7C) and ESTIMATE score (Figure 7E) were significantly higher in the high VM score group than in the low VM score group, while there was no difference in the immune score (Figure 7D), which demonstrated a worse immune response in the high VM score group. The above results suggested that the VM score was a good indicator for evaluating immune cell infiltration in patients with lung adenocarcinoma and that the VM score may be a potential biomarker to guide immunotherapy for lung adenocarcinoma patients.

### 3.7 Immune checkpoints and drug sensitivity associations with the VM score

**FIGURE 8:**
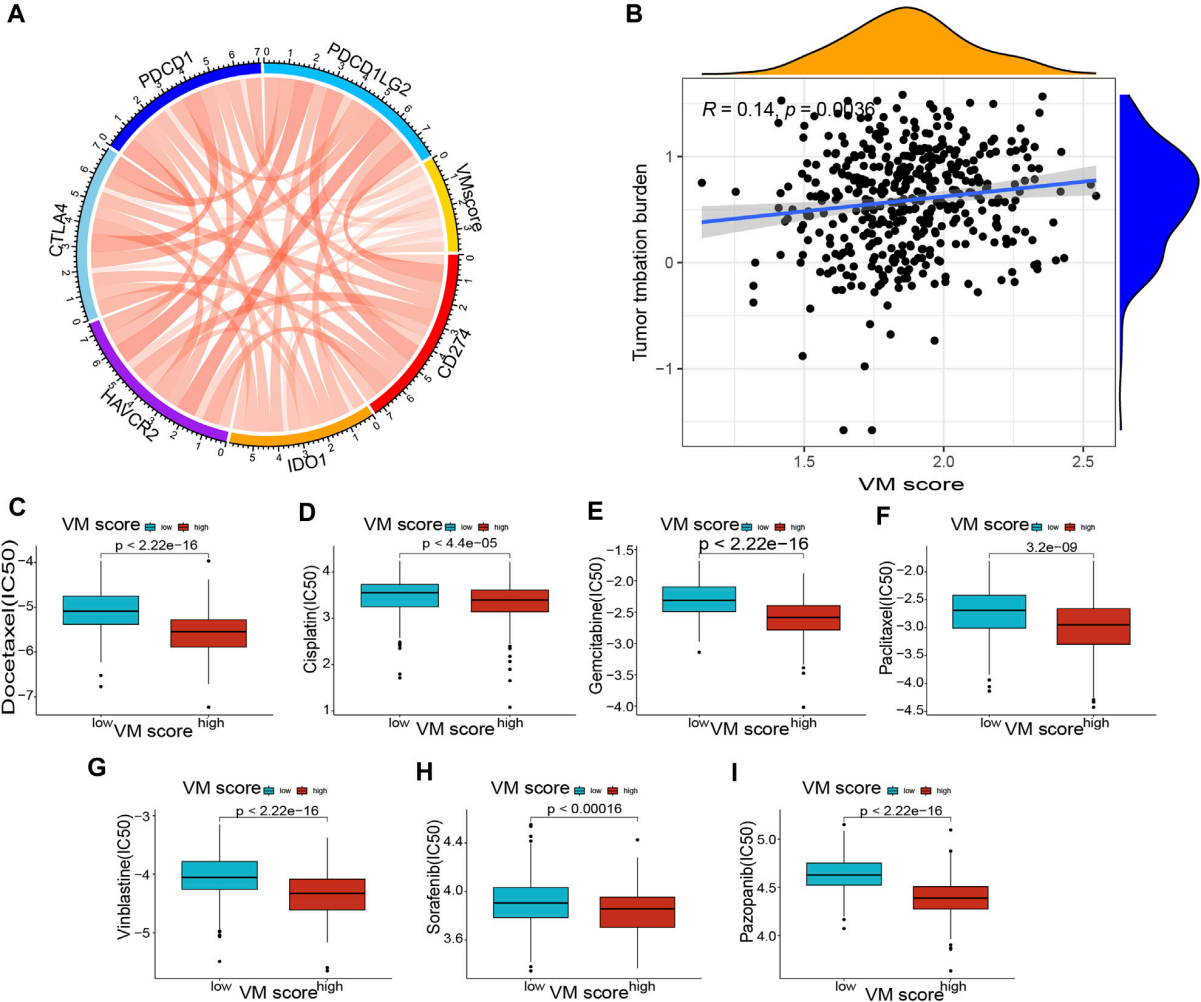
Correlation of VM score with immune checkpoints and drug sensitivity. **(A)** Correlation of VM score with immune checkpoints. **(B)** Correlation of VM score with TMB. **(C–I)**: Correlation of VM score with drug sensitivity. **(C)** Docetaxel, **(D)** Cisplatin, **(E)** Gemcitabine, **(F)** Paclitaxel, **(G)** Vinblastine, **(H)** Sorafenib, **(I)** Pazopanib. VM: vasculogenic mimicry. TMB: tumor mutation burden.

The previous results suggested that the VM score may be a potential biomarker for the evaluation of immunotherapy. To further evaluate the correlation of the VM score with immunotherapy, we explored the correlation between immune checkpoints and the VM score. The results showed that the expression of CD274, IDO1, HAVCR2, CTLA4, PDCD1, and PDCD1LG2 was positively correlated with the VM score (Figure 8A), which suggested that a high VM score predicts immune checkpoint activity and reduces the effect of immune checkpoint blockade (ICB). The results showed that the VM score was positively correlated with TMB (Figure 8B), which indicated that tumor mutations were more frequent in patients with a high VM score. Additionally, we tested the difference in drug sensitivity between the high VM score group and the low VM score group. The results showed that the IC50 values of docetaxel (Figure 8C), cisplatin (Figure 8D), gemcitabine (Figure 8E), paclitaxel (Figure 8F), vinblastine (Figure 8G), sorafenib (Figure 8H) and pazopanib (Figure 8I) were lower in the high VM score group (*p* < 0.05), suggesting better affinity of the above drugs in the high VM score group. These results demonstrated that the VM score may be a potential indicator for assessing immunotherapy in patients with lung adenocarcinoma.

## 4 Discussion

Lung cancer remains a serious health hazard and the leading cause of cancer deaths in China (Wu et al., 2021). LUAD is the most common type of lung cancer, accounting for approximately 40% of all types (Siegel et al., 2019). The advent of immunotherapy and targeted therapies has disrupted the classic platinum-containing treatments for lung cancer; unfortunately, drug resistance remains an inevitable problem (Lim and Ma, 2019). VEGF is the main signaling pathway for tumor angiogenesis, but antiangiogenic drugs targeting VEGF have not achieved the desired results (Ren et al., 2021). VM is another modality of tumor angiogenesis that has the potential to be a target for antitumor therapy (Luo et al., 2020). Previous studies confirmed that HIF-1α, VEGF, Wnt/β-catenin, and MAPK are possible signaling pathways for VM; however, their specific roles in VM formation are not clear (Wechman et al., 2020). Wang et al. (2022) evaluated the prognostic value of VM-related genes and demonstrated that VM may be a potential target for evaluating immunotherapy in gastric cancer. Previous studies confirmed the regulatory effects of ARHGAP25, RhoA, PP2A, MMP2, and sema4D on VM based on lung cancer cells (Li et al., 2017; Xia et al., 2019; Shi et al., 2022; Zhang et al., 2022), suggesting the involvement of these molecules in the VM formation process. Here, we identified VM-related hub genes associated with the prognosis of LUAD patients and developed a VM score based on 3 prognostic genes (EPHA2, LAMC2, and LOXL2). We confirmed that a nomogram based on the VM score could predict the survival probability of LUAD patients; in addition, the VM score has important value in assessing the immune landscape immunotherapy response in LUAD patients.

In the hypoxic microenvironment, the HIF signaling pathway induces cancer stem cell (CSC) differentiation, and CSCs acquire endothelial-like features and eventually participate in VM (Wei et al., 2021). Our study also identified VM-related genes significantly enriched in the HIF-1 signaling pathway, suggesting a critical role of HIF-1 in the VM process. In addition, PGs in cancer, the VEGF signaling pathway and the relaxin signaling pathway are also pathways of VM-related genes. PGs are components of the extracellular matrix (ECM) that promote tumor angiogenesis and provide favorable conditions for tumor invasion and metastasis (Wei et al., 2020). VEGF is a major effector of tumor angiogenesis, and VEGF induces VM formation in melanoma through activation of the PI3K/Akt signaling pathway (Xu et al., 2019). A recent review suggested that the process of tumor development is a multidimensional ecological disease; VM is formed to adopt multiple cellular phenotypes due to the external environment, which contributes to understand the mechanism of VM formation in lung cancer (Luo, 2023). Collectively, the results of the enrichment analysis showed that the VM-related signaling pathways were the VEGF signaling pathway, HIF-1 signaling pathway and PGs in cancer, which is consistent with previous studies (Xu et al., 2019; Wei et al., 2020).

The vascular lining structure in VM is devoid of endothelial cells, and the formation of VM is not dependent on vascular endothelial cells (Xiang et al., 2018). Therefore, tumor cells are prone to metastasize through the bloodstream under this condition, which also suggests that VM is often found in highly aggressive and metastatic tumors and is associated with a poor prognosis (Yang et al., 2016). Our results demonstrated that the VM score is associated with poor prognosis in LUAD patients based on EPHA2, LAMC2, and LOXL2. EPHA2, a receptor tyrosine kinase, promotes the proliferation and migration of lung cancer cells and is associated with poor prognosis of lung cancer (Ishikawa et al., 2012). Wang et al. reported that EPHA2 contributes to VM in prostate cancer and could be a potential therapeutic target (Wang et al., 2016). LAMC2, a member of the extracellular matrix glycoproteins, is an important part of the epithelial basement membrane (Moon et al., 2015). LAMC2 promotes the migration and invasion of LUAD cells and is associated with LUAD prognosis (Moon et al., 2015). Subsequently, Okada et al. identified that LAMC2 not only correlates with OS in pancreatic cancer but also modulates gemcitabine sensitivity (Okada et al., 2021), suggesting that LAMC2 is a promising target in pancreatic cancer. LOXL2, a member of the lysyl oxidase (LOX) family, regulates epithelial-mesenchymal transition (EMT) through the Snail pathway. It was found that LOXL2 is highly expressed in tumor cells and promotes tumor cell invasion and metastasis (Wu and Zhu, 2015). The function of EPHA2, LAMC2 and LOXL2 in tumor cells may explain the correlation between the VM score and poor prognosis of LUAD patients. Previous studies have also demonstrated that the presence of VM is associated with poor prognosis of tumors, such as in patients with gastric cancer, melanoma, hepatocellular carcinoma, and breast cancer (Luo et al., 2020). VM correlated with clinical parameters (tumor size, lymph node metastasis, and poor differentiation) (Jafarian et al., 2019), indicating that the presence of VM is often found at an advanced stage of the tumor. Our results indicated that the VM score is associated with worsening T stage, which also indicated that high expression of VM-related genes is associated with poor prognosis in LUAD patients. Overall, the VM score was a risk factor for LUAD patients and was associated with poor prognosis in LUAD patients. In addition, a nomogram constructed by combining clinical information could realistically predict the survival probability of LUAD patients.

The TME and immune cell infiltration play key roles in tumor development and the tumor immune response (Mao et al., 2021). We demonstrated that the VM score is a valuable indicator to assess immune cell infiltration in LUAD patients. The expression of naïve B cells, activated memory CD4 T cells, follicular helper T cells, activated NK cells, eosinophils, and neutrophils was significantly correlated with the VM score based on the CIBERSORT results. We discovered that neutrophil levels were positively correlated with the VM score, suggesting that neutrophils may promote tumor growth, while neutrophils were reported to have both promoting and inhibiting effects on tumor growth (Quail et al., 2022). Naïve B cells, a member of the TME, inhibit tumor growth (Zhang et al., 2021), and we discovered a negative correlation between naïve B cells and the VM score, suggesting that VM may be a factor in promoting tumor growth. In addition, we confirmed that the TME score in the high VM score group was higher than that in the low VM score group (stromal score and ESTIMATE score). The TME plays an important role not only in tumor development and metastasis but also in tumor treatment response and drug resistance (Wu and Dai, 2017). In fact, the purpose of the TME is mainly to safeguard the growth of tumor cells. In the hypoxic and acidic TME, tumor cells need to obtain oxygen and nutrients through VM (Andreucci et al., 2022), which may explain the correlation between the TME and VM scores. Our study confirmed the importance of the VM score in assessing the TME in LUAD patients. The evaluation of immune checkpoints is a critical step in immunotherapy for LUAD patients (Li et al., 2019). We identified a positive correlation between the VM score and the expression of common immune checkpoint genes (CD274, IDO1, HAVCR2, CTLA4, PDCD1 and PDCD1LG2), which suggested that LUAD patients with a high VM score may be more sensitive to immunotherapy. The VM score may be a valuable indicator for assessing the response to immunotherapy in LUAD patients. Finally, we found that patients with high VM scores were more sensitive to common antitumor drugs (docetaxel, cisplatin, gemcitabine, paclitaxel and vinblastine) than those with low VM scores. Of note, we screened two antiangiogenic drugs, pazopanib and sorafenib, which may be more suitable for high VM score patients with LUAD. We confirmed that the VM score is a valuable indicator for assessing the immune landscape of LUAD patients. Furthermore, the VM score was significantly correlated with the expression levels of immune checkpoint-related genes, and patients with high VM score LUAD were more sensitive to immunotherapy and antiangiogenic drugs.

The VM score is a valuable indicator in evaluating the prognosis and immune profile of LUAD patients; however, there are several limitations. First, our study was retrospective and subject to selection bias. Second, we failed to confirm the relationship between VM score and immune cell infiltration in lung cancer tissues. Third, our study was not able to complete *in vivo* or *in vitro* experiments to confirm the specific function of VM-related genes in lung cancer. These are the directions of our future research.

## 5 Conclusion

In conclusion, we developed a VM score based on EPHA2, LAMC2 and LOXL2 in LUAD patients. We confirmed that VM may be a valuable indicator to evaluate the prognosis, immune cell infiltration and TME of LUAD patients and can contribute to guiding immunotherapy.

## Data Availability

The original contributions presented in the study are included in the article/Supplementary Material, further inquiries can be directed to the corresponding author.
