# Supplementary material for: Vasculogenic mimicry score identifies the prognosis and immune landscape of lung adenocarcinoma
Source: Front Genet. 2023 Jun 7;14:1206141. doi: 10.3389/fgene.2023.1206141 (PMC10282128; doi:10.3389/fgene.2023.1206141)
Supplement: Supplementary file 1 [file Table1.DOCX]

Supplementary Material

Vasculogenic mimicry score identifies the prognosis and immune landscape of lung adenocarcinoma

**Weichang Yang^1^, Zhouhua Li ^1^, Wenjun Wang ^1^, Juan Wu^1^, Jinbo Li^1^, Xiaotian Huang ^1^, Xinyi Zhang^1^ and Xiaoqun Ye^1^***

*** Correspondence:** Xiaoqun Ye: 511201663@qq.com

# Supplementary Tables

**Table S1.** The literature sources for 24 VM-related genes

| TFPI | Ruf W, Seftor EA, Petrovan RJ, et al. Differential role of tissue factor pathway inhibitors 1 and 2 in melanoma vasculogenic mimicry. Cancer Res. 2003;63(17):5381-5389. |
| --- | --- |
| SERPINF1, LOXL2 | Lee M, Cho HJ, Park KS, Jung HY. ELK3 Controls Gastric Cancer Cell Migration and Invasion by Regulating ECM Remodeling-Related Genes. Int J Mol Sci. 2022;23(7):3709. Published 2022 Mar 28. doi:10.3390/ijms23073709 |
| TF | Yu J, May L, Milsom C, et al. Contribution of host-derived tissue factor to tumor neovascularization. Arterioscler Thromb Vasc Biol. 2008;28(11):1975-1981. doi:10.1161/ATVBAHA.108.175083 |
| MAPK1, MAPK3 | Herrera-Vargas AK, García-Rodríguez E, Olea-Flores M, Mendoza-Catalán MA, Flores-Alfaro E, Navarro-Tito N. Pro-angiogenic activity and vasculogenic mimicry in the tumor microenvironment by leptin in cancer. Cytokine Growth Factor Rev. 2021;62:23-41. doi:10.1016/j.cytogfr.2021.10.006 |
| PIK3CA, KDR | Zhu Y, Liu X, Zhao P, Zhao H, Gao W, Wang L. Celastrol Suppresses Glioma Vasculogenic Mimicry Formation and Angiogenesis by Blocking the PI3K/Akt/mTOR Signaling Pathway. Front Pharmacol. 2020;11:25. Published 2020 Feb 6. doi:10.3389/fphar.2020.00025 |
| ROCK1, ROCK2 | Zhang JG, Zhou HM, Zhang X, et al. Hypoxic induction of vasculogenic mimicry in hepatocellular carcinoma: role of HIF-1 α, RhoA/ROCK and Rac1/PAK signaling. BMC Cancer. 2020;20(1):32. Published 2020 Jan 13. doi:10.1186/s12885-019-6501-8 |
| VEGFA, CDH5, SNAI1, SNAI2 | Sun D, Sun B, Liu T, et al. Slug promoted vasculogenic mimicry in hepatocellular carcinoma. J Cell Mol Med. 2013;17(8):1038-1047. doi:10.1111/jcmm.12087 |
| NOTCH1 | Wechman SL, Emdad L, Sarkar D, Das SK, Fisher PB. Vascular mimicry: Triggers, molecular interactions and in vivo models. Adv Cancer Res. 2020;148:27-67. doi:10.1016/bs.acr.2020.06.001 |
| EPHA2 | Kim HS, Won YJ, Shim JH, et al. Morphological characteristics of vasculogenic mimicry and its correlation with EphA2 expression in gastric adenocarcinoma. Sci Rep. 2019;9(1):3414. Published 2019 Mar 4. doi:10.1038/s41598-019-40265-7 |
| PTGS2, MMP9 | Liu XM, Zhang QP, Mu YG, et al. Clinical significance of vasculogenic mimicry in human gliomas. J Neurooncol. 2011;105(2):173-179. doi:10.1007/s11060-011-0578-5 |
| TWIST1, TGFB1, TWIST2, LAMC2 | Morales-Guadarrama G, García-Becerra R, Méndez-Pérez EA, García-Quiroz J, Avila E, Díaz L. Vasculogenic Mimicry in Breast Cancer: Clinical Relevance and Drivers. Cells. 2021;10(7):1758. Published 2021 Jul 12. doi:10.3390/cells10071758 |
| MMP2 | Lu XS, Sun W, Ge CY, Zhang WZ, Fan YZ. Contribution of the PI3K/MMPs/Ln-5γ2 and EphA2/FAK/Paxillin signaling pathways to tumor growth and vasculogenic mimicry of gallbladder carcinomas. Int J Oncol. 2013;42(6):2103-2115. doi:10.3892/ijo.2013.1897 |
| TFPI2 | Mo J, Zhao X, Wang W, et al. TFPI2 Promotes Perivascular Migration in an Angiotropism Model of Melanoma. Front Oncol. 2021;11:662434. Published 2021 Jun 24. doi:10.3389/fonc.2021.662434 |

**Table S2.** GO enrichment analysis of VM-related genes

| Category | ID | Description | pvalue | geneID |
| --- | --- | --- | --- | --- |
| BP | GO:0001667 | ameboidal-type cell migration | 6.44144E-17 | SERPINF1/PIK3CA/ROCK1/VEGFA/NOTCH1/ROCK2/EPHA2/CDH5/KDR/PTGS2/MMP9/TWIST1/LOXL2/TGFB1 |
|  | GO:0010631 | epithelial cell migration | 7.20456E-17 | SERPINF1/PIK3CA/ROCK1/VEGFA/NOTCH1/ROCK2/EPHA2/CDH5/KDR/PTGS2/MMP9/LOXL2/TGFB1 |
|  | GO:0090132 | epithelium migration | 8.03169E-17 | SERPINF1/PIK3CA/ROCK1/VEGFA/NOTCH1/ROCK2/EPHA2/CDH5/KDR/PTGS2/MMP9/LOXL2/TGFB1 |
|  | GO:0090130 | tissue migration | 9.6068E-17 | SERPINF1/PIK3CA/ROCK1/VEGFA/NOTCH1/ROCK2/EPHA2/CDH5/KDR/PTGS2/MMP9/LOXL2/TGFB1 |
|  | GO:0003180 | aortic valve morphogenesis | 6.94244E-15 | ROCK1/NOTCH1/ROCK2/SNAI1/TWIST1/SNAI2/TGFB1 |
|  | GO:0043542 | endothelial cell migration | 1.32879E-14 | SERPINF1/PIK3CA/VEGFA/NOTCH1/ROCK2/EPHA2/CDH5/KDR/PTGS2/LOXL2/TGFB1 |
|  | GO:0003176 | aortic valve development | 2.11515E-14 | ROCK1/NOTCH1/ROCK2/SNAI1/TWIST1/SNAI2/TGFB1 |
|  | GO:0001503 | ossification | 2.80731E-14 | MAPK1/NOTCH1/MAPK3/EPHA2/PTGS2/MMP9/SNAI1/TWIST1/MMP2/SNAI2/TGFB1/TWIST2 |
|  | GO:1905314 | semi-lunar valve development | 4.60418E-14 | ROCK1/NOTCH1/ROCK2/SNAI1/TWIST1/SNAI2/TGFB1 |
|  | GO:0048762 | mesenchymal cell differentiation | 1.88596E-13 | MAPK1/ROCK1/NOTCH1/ROCK2/MAPK3/SNAI1/TWIST1/LOXL2/SNAI2/TGFB1 |
| CC | GO:0005901 | caveola | 2.84014E-06 | TFPI/MAPK1/MAPK3/PTGS2 |
|  | GO:0044853 | plasma membrane raft | 1.01936E-05 | TFPI/MAPK1/MAPK3/PTGS2 |
|  | GO:0062023 | collagen-containing extracellular matrix | 1.0245E-05 | SERPINF1/LAMC2/MMP9/MMP2/LOXL2/TGFB1 |
|  | GO:0034774 | secretory granule lumen | 3.82023E-05 | TF/MAPK1/ROCK1/VEGFA/TGFB1 |
|  | GO:0060205 | cytoplasmic vesicle lumen | 3.99294E-05 | TF/MAPK1/ROCK1/VEGFA/TGFB1 |
|  | GO:0045121 | membrane raft | 4.05184E-05 | TFPI/MAPK1/MAPK3/KDR/PTGS2 |
|  | GO:0031983 | vesicle lumen | 4.11141E-05 | TF/MAPK1/ROCK1/VEGFA/TGFB1 |
|  | GO:0098857 | membrane microdomain | 4.11141E-05 | TFPI/MAPK1/MAPK3/KDR/PTGS2 |
|  | GO:0005604 | basement membrane | 0.000207552 | SERPINF1/LAMC2/LOXL2 |
|  | GO:0031143 | pseudopodium | 0.000217373 | MAPK1/MAPK3 |
| MF | GO:0004712 | protein serine/threonine/tyrosine kinase activity | 1.13177E-06 | MAPK1/PIK3CA/ROCK1/ROCK2/MAPK3/EPHA2/KDR |
|  | GO:0070888 | E-box binding | 3.44794E-05 | SNAI1/TWIST1/SNAI2 |
|  | GO:0004866 | endopeptidase inhibitor activity | 8.05922E-05 | TFPI/SERPINF1/ROCK1/TFPI2 |
|  | GO:0106310 | protein serine kinase activity | 8.70175E-05 | MAPK1/PIK3CA/ROCK1/ROCK2/MAPK3 |
|  | GO:0030414 | peptidase inhibitor activity | 9.34267E-05 | TFPI/SERPINF1/ROCK1/TFPI2 |
|  | GO:0061135 | endopeptidase regulator activity | 0.000107689 | TFPI/SERPINF1/ROCK1/TFPI2 |
|  | GO:0004857 | enzyme inhibitor activity | 0.000126781 | TFPI/SERPINF1/ROCK1/NOTCH1/TFPI2 |
|  | GO:0005172 | vascular endothelial growth factor receptor binding | 0.000146806 | VEGFA/CDH5 |
|  | GO:0004707 | MAP kinase activity | 0.000169257 | MAPK1/MAPK3 |
|  | GO:0004674 | protein serine/threonine kinase activity | 0.000200004 | MAPK1/PIK3CA/ROCK1/ROCK2/MAPK3 |

**Table S3.** Top pathways from KEGG enrichment based on VM-related genes

| ID | Description | pvalue | geneID |
| --- | --- | --- | --- |
| hsa05205 | Proteoglycans in cancer | 2.08174E-14 | MAPK1/PIK3CA/ROCK1/VEGFA/ROCK2/MAPK3/KDR/MMP9/TWIST1/MMP2/TGFB1/TWIST2 |
| hsa04370 | VEGF signaling pathway | 7.0251E-09 | MAPK1/PIK3CA/VEGFA/MAPK3/KDR/PTGS2 |
| hsa04926 | Relaxin signaling pathway | 2.71673E-08 | MAPK1/PIK3CA/VEGFA/MAPK3/MMP9/MMP2/TGFB1 |
| hsa04510 | Focal adhesion | 2.7777E-08 | MAPK1/PIK3CA/ROCK1/VEGFA/ROCK2/MAPK3/LAMC2/KDR |
| hsa05219 | Bladder cancer | 5.8207E-08 | MAPK1/VEGFA/MAPK3/MMP9/MMP2 |
| hsa01522 | Endocrine resistance | 1.53753E-07 | MAPK1/PIK3CA/NOTCH1/MAPK3/MMP9/MMP2 |
| hsa04933 | AGE-RAGE signaling pathway in diabetic complications | 1.73537E-07 | MAPK1/PIK3CA/VEGFA/MAPK3/MMP2/TGFB1 |
| hsa04670 | Leukocyte transendothelial migration | 3.79242E-07 | PIK3CA/ROCK1/ROCK2/CDH5/MMP9/MMP2 |
| hsa05206 | MicroRNAs in cancer | 7.31035E-07 | MAPK1/PIK3CA/ROCK1/VEGFA/NOTCH1/MAPK3/PTGS2/MMP9 |
| hsa05211 | Renal cell carcinoma | 8.31785E-07 | MAPK1/PIK3CA/VEGFA/MAPK3/TGFB1 |
